# Validation of activity questionnaires in patients with cystic fibrosis by accelerometry and cycle ergometry

**DOI:** 10.1186/1471-2288-12-43

**Published:** 2012-04-03

**Authors:** Katharina C Ruf, Sonja Fehn, Michèle Bachmann, Alexander Moeller, Kristina Roth, Susi Kriemler, Helge Hebestreit

**Affiliations:** 1 Department of Pediatrics, University of Wuerzburg, Josef-Schneider-Strasse 2, Würzburg, Germany; 2 Division of Respiratory Medicine, University Children's Hospital, University of Zurich, Steinwiesstrasse 75, Zürich, Switzerland; 3 Swiss Tropical and Public Health Institute, University of Basel, Socinstrasse 57, Basel, Switzerland

## Abstract

**Background:**

The objective of this study was to validate physical activity questionnaires for cystic fibrosis (CF) against accelerometry and cycle ergometry.

**Methods:**

41 patients with CF (12-42 years) completed the Habitual Activity Estimation Scale (HAES), the 7-Day Physical Activity Recall questionnaire (7D-PAR) and the Lipid Research Clinics questionnaire (LRC) and performed an incremental exercise test according to the Godfrey protocol up to volitional fatigue. Time spent in moderate and vigorous physical activity (MVPA) assessed objectively by accelerometry was related to the time spent in the respective activity categories by correlation analyses and calculating intraclass correlation coefficients (ICC). Furthermore, the results of the exercise test were correlated with the results of the questionnaires.

**Results:**

Time spent in the categories 'hard','very hard' and 'hard & very hard' of the 7D-PAR (0.41 < r < 0.56) and 'active' (r = 0.33) of the HAES correlated significantly with MVPA. The activity levels of the LRC were not related to objectively determined physical activity. Significant ICCs were only observed between the 7D-PAR activitiy categories and MVPA (ICC = 0.40-0.44). Only the LRC showed moderate correlations with the exercise test (Wmax: r = 0.46, *p *= 0.002; VO_2_peak: r = 0.32, *p *= 0.041).

**Conclusions:**

In conclusion, the activity categories 'hard' and 'very hard' of the 7D-PAR best reflected objectively measured MVPA. Since the association was at most moderate, the 7D-PAR may be selected to describe physical activity within a population. None of the evaluated questionnaires was able to generate valid physical activity data exercise performance data at the individual level. Neither did any of the questionnaires provide a valid assessment of aerobic fitness on an invidual level.

## Background

Regular physical activity and the training of aerobic fitness have become part of the treatment of cystic fibrosis (CF) because they contribute to a slower decline in lung function, a better nutritional status as well as an improved quality of life [1-3]. Furthermore, physical activity and aerobic fitness are related [4] and a high aerobic fitness has been linked to survival in CF [5].

Despite the importance of physical activity for patients' health and quality of life in CF, the amount and intensities of the patients' activities are not regularly assessed in the clinical setting and physical activity has not yet been incorporated in CF registries. This is probably due to the fact that there are almost no validated tools to quickly, easily and reliably assess the level of physical activity in the CF population.

In general, the assessment of physical activity is hampered by difficulties to accurately determine and recall the type and intensity of all possible activities. Accelerometry has therefore been used over the last years to objectively measure physical activity levels and patterns in the healthy population and in patients with CF [1,4,6], and has widely been accepted as a valid tool to assess physical activity [7] Likewise, peak oxygen uptake (VO_2_peak) as a measure of aerobic fitness has been used as surrogate measure of PA [8]. Yet, accelerometry and cycle ergometry are expensive and time consuming and require specialized equipment. For the routine assessment of patients' physical activity in a clinical setting, a quick and easily accessible tool at low costs is required. Therefore, questionnaires might be best suited to assess physical activity. Some of the existing instruments such as the Lipid Research Clinics (LRC), the Seven Day Physical Activity Recall (7D-PAR), and the Habitual Activity Estimation Scale (HAES) questionnaire have been used and validated in healthy children and adults [9-12] and have also been employed in patients with CF [2,13,14]. However, in patients with CF a validation against objective measures of physical activity has only been performed for the HAES in a relatively small group of young patients [13] and to our knowledge a validation of questionnaires with respect to aerobic fitness has not been performed at all.

Therefore, the objective of this study was to validate commonly used questionnaires for the assessment of physical activity (HAES, 7D-PAR and LRC) in a larger group of patients with CF including children and adults against accelerometry (to assess physical activity behaviour) and cycle ergometry (to assess aerobic fitness).

## Methods

### Participants

In 2006, 41 participants with a proven diagnosis of CF (12-to 42-years old, 11 adult participants) were recruited from the CF centre at the University Children's Hospital of Wuerzburg, Germany (13 female, 8 male) and from the CF centre at the Children's Hospital of Zurich, Switzerland (10 female, 10 male). All patients of the respective centres with an age of 12 years or higher and a proven diagnosis of CF were invited to participate in the study. The diagnosis of CF was based on CF-typical symptoms and clinical findings and either two pathological sweat tests or the discovery of CF-relevant mutations in both alleles of the CFTR gene. Patients with multiresistant bacteria and acute exacerbation at the time of assessments as defined by published criteria [15] were excluded from the study. The local ethics committee approved the study protocol. Written informed consent of the participants and, if applicable, their legal guardians was obtained after explaining the study procedures to the participants.

### Study design

Participants who agreed to take part in the study during a regular clinical visit wore an accelerometer for 7 consecutive days. Two to six weeks later, the participants returned to the respective CF centres for anthropometric measurements, a lung function test, an incremental exercise test on a cycle ergometer and the completion of the HAES, 7D-PAR and LRC physical activity questionnaires. In a convenient subgroup of 19 participants, accelerometry was repeated four to six weeks after the first assessment to determine consistency in activity behaviour.

### Procedures

#### Anthropometry and lung function testing

On the day of testing, height and weight were determined in light clothing without shoes. Pulmonary function and lung volumes were assessed by spirometry and bodyplethysmography, respectively (in German subjects by Masterscreen Body, Jaeger, Wuerzburg, Germany; in Swiss subjects by Masterlab, Jaeger, Wuerzburg, Germany). Forced expiratory volume in one second (FEV1) and forced vital capacity (FVC) are reported as %predicted [16]. Residual volume (RV) is expressed in % of total lung capacity (TLC).

### Assessments of activity behaviour

#### Accelerometry

The participants wore an accelerometer for 7 days on their right hip (GT1M, ActiGraph, Pensacola, FL, USA). Epoch time was set to 60 s as has been employed by Hebestreit et al. before [4,6]. If the participants' data included intervals of zero activity for 10 minutes or longer, the time period with zero readings was removed from the data [17]. Participants who did not complete at least three days of valid recording, including one weekend day, with at least 9 hours of valid data each day, were excluded from further analysis [17]. Due to the fact that there are no validated cut-offs for different activity levels in CF, we decided to use the cut-offs that had been used before by Hebestreit et al. [4,6]., Thus, moderate and vigorous physical activity (MVPA) was defined as time spent in an activity level of at least 1000 counts per minute, moderate physical activity (MPA) was classified between 1000 and 1999 counts per minute, and vigorous physical activity (VPA) was assumed if participants achieved 2000 or more counts per minute. These cut-offs were based on the following reasoning: In most validation studies in a healthy population, validation was performed through activities like running and walking which are well represented by accelerometers. For such activities, cut-offs around 2000 counts per minute have been published to discriminate light from moderate intensities [18,19]. However, if a wide range of activities was included in the validation process, a value of 191 counts per minute has been reported as a cut-off between light and moderate activities [18].

Time spent in each of the activity levels was determined separately for weekdays and for weekend days. To determine average MVPA, MPA, and VPA per day, the weekdays' average was taken times 5, summed up with the weekend days' average times 2 and divided by 7.

#### Questionnaires

As the questionnaires did not exist in a German version and all participants were German native speakers, the 7D-PAR, the HAES, and the LRC [8,11,12] were all translated into German by H Hebestreit. They were retranslated into English by S Kriemler and were checked by native speakers to ensure a correct translation. The German version for the 7D-PAR and the LRC had used in the past [20], while the German version of the HAES was first used in this study. For the 7D-PAR, times spent in the activity categories 'moderate', 'hard', and 'very hard' were calculated. Likewise, for the HAES times spent in categories 'somewhat active' and 'active' were computed. For the LCR, an activity level was derived for each individual (1 - very low active to 4- high active). All questionnaires have been validated in a test-retest analysis before. For the 7D-PAR, Sallis et al. administered the questionnaire three times during the validation process to check for reliability and found a highly significant Pearson correlation coefficient of r = 0.75 for moderate and r = 0.85 for vigorous activities [11]. In a sample of children and adolescents, they found a test-retest reliability of r = 0.77 [10]. For the LRC, Ainsworth et al. administered the *questionnaire two times and found a correlation coefficient of r = 0.85 *[8]. The test-retest reliability of the HAES was analyzed by Wells et al. in patients with CF who found a highly significant intra-class correlation coefficient ICC = 0.72 [13].

Further details regarding the questionnaires are provided in the additional material (Additional file 1).

### Assessment of aerobic fitness

After familiarizing the patient with the cycle ergometer (Ergomedic 834 E, Monark, Sweden for the German subjects and Ergometrics 900, Ergoline, Bitz, Germany for the Swiss subjects) and the gas sampling equipment, an incremental exercise test was performed according to the Godfrey protocol [21]. Work rate was set depending on the height of the patient: patients with a height between 120 and 150 cm started with 15 W, patients taller than 150 cm started with 20 W. Work load was increased minute-by-minute by 15 W or 20 W, respectively, up to volitional fatigue. Physical working capacity was determined as the highest work rate performed for one minute and expressed in % predicted [21]. During the exercise test, ventilation and gas exchange data were recorded breath-by-breath using a metabolic cart, and averaged every 15 seconds (German patients: CPX/D, MedGraphics, St. Paul, MN, USA; Swiss patients: Cortex MetaLyzer, Metamax CORTEX Biophysik GmbH, Leipzig, Germany). Peak oxygen uptake (VO_2_peak) was taken as the highest oxygen uptake over two consecutive 15-s intervals during the test and expressed in % predicted [22]. Maximal power (Wmax) was defined as the highest workload that could be maintained for a minute.

### Statistical analysis

Student-t tests were used to test for differences between German and Swiss participants, and between female and male participants with respect to age, anthropometrical data, pulmonary function measures, lung volumes, exercise performance and physical activity as all variables were normally distributed. All variables of the accelerometry and the questionnaires (except for the LRC) that assessed time within a certain activity level were expressed in minutes per day. The time spent in certain activity levels derived from the 7D-PAR and HAES questionnaires were related to the time spent in MPA, VPA and MVPA measured objectively by accelerometry as well as to exercise performance using Pearson product correlation coefficients. Associations between individual activity levels determined from the LRC and physical activity assessed by accelerometry as well as exercise performance were analysed using Spearman rank correlations. Validity of the HAES and the 7D-PAR was further tested calculating intraclass correlations (ICC) with the accelerometer data. ICC analyses were used to assess test-retest reliability in the subgroup of participants with two accelerometry recording periods with 4 to 6 weeks apart. Furthermore, limits of agreement were calculated in addition to ICC analyses and the data graphically displayed by a Bland-Altman plot.

All statistical analyses were performed using SPSS 17 (SPSS Inc., Chicago, IL), statistical significance was assumed at *p *< 0.05.

## Results

Data for the participants of the two countries were pooled, since there were no significant differences between the groups for the physical activity variables. Table 1 summarizes the characteristics of the participants. No significant differences were found between male and female participants. The Swiss participants were significantly younger and lighter and showed a significantly lower FVC in %predicted than the German participants (mean age: Swiss participants 14.7 years, German participants 18.6 years, *p *= .023; mean weight: Swiss participants 47.6 kg, German participants 54.8 kg, *p *= .046; mean FVC: Swiss participants 80.3%predicted, German participants 96.0%predicted, *p *= .010) but did not show differences in activity measures.

**Table 1:** Patients' characteristics

	Male patients (n = 18; 4 adults)	Female patients (n = 23; 7 adults)
Age (years)	15.9 ± 4.5	17.4 ± 6.4

Height (cm)	161.7 ± 10.4	159.1 ± 7.1

Weight (kg)	50.8 ± 12.1	51.7 ± 2.4

FVC (%predicted)	86.8 ± 15.1	89.6 ± 23.2

FEV1 (%predicted)	76.3 ± 20.5	78.0 ± 5.8

RV/TLC (%)	29.5 ± 14.2	34.7 ± 15.5

Values are means ± standard deviations.

Table 2 summarizes the physical activity determined by accelerometry and the HAES and 7D-PAR questionnaires as well as the results of the exercise test.

**Table 2:** Time spent in certain physical activity categories as derived from questionnaires and accelerometry, and physical fitness as determined by cycle ergometry for male and female participants of the study

Instrument	Variable	Male patients (n = 18; 4 adults)	Female patients (n = 23; 7 adults)
Physical activity			

7D-PAR	moderate (min/d)	101.1 ± 117.6	114.7 ± 126.1
	
	hard (min/d)	48.1 ± 60.8	50.9 ± 68.8
	
	very hard (min/d)	38.5 ± 51.8	20.3 ± 26.8
	
	Moderate & hard & very hard (min/d)	187.7 ± 193.2	185.8 ± 177.1
	
	hard & very hard (min/d)	86.6 ± 102.3	71.2 ± 77.4

HAES	active (min/d)	137.0 ± 132.2	136.5 ± 137.6
	
	somewhat active & active (min/d)	358.8 ± 185.6	445.3 ± 202.5

Accelerometry	MPA (min/d)	49.5 ± 17.8	42.1 ± 29.7
	
	VPA (min/d)	47.7 ± 24.7	28.6 ± 14.1 **
	
	MVPA (min/d)	97.2 ± 36.5	70.8 ± 35.4 *

Physical fitness			

Cycle ergometry	Wmax (% predicted)	112.5 ± 22.1	103.5 ± 15.1
	
	VO_2_peak (% predicted)	88.5 ± 16.3	81.0 ± 13.3

Values are mean ± standard deviation.

Abbreviations: 7D-PAR - seven day physical activity recall questionnaire; HAES - habitual activity estimation scale; min/d - minutes per day; MPA - moderate physical activity; VPA - vigorous physical activity; MVPA - moderate and vigorous physical activity; Wmax - maximal power during cycle ergometry; VO2peak - peak oxygen uptake.

While there was no gender difference in physical activity assessed by the questionnaires, males spent significantly more time than females in the accelerometer categories VPA and MVPA, but not MPA.

Employing the LRC, 3 males and 6 females were classified as 'very low active', 5 males and 9 females as 'low active', 8 males and 8 females as 'moderately active', and 2 males but no female as 'very active'.

Table 3 summarizes the relationships between physical activity as determined by accelerometry and physical activity estimated from questionnaires.

**Table 3:** Associations between objectively measured physical activity and physical activity assessed by questionnaires

		MPA (min/d)	VPA (min/d)	MVPA (min/d)
7D-PAR	moderate (min/d)	r = .167 p = .298	r = .-038 p = .816	r = .090 p = .577
	
	hard (min/d)	**r = .661** **p = .000**	r = .118 p = .461	**r = .508** **p = .001**
	
	very hard (min/d)	**r = .340, p = .030**	**r = .321, p = .041**	**r = .409, p = .008**
	
	Moderate & hard & very hard (min/d)	**r = .421, p = .006**	r = .088, p = .584	**r = .330, p = .035**
	
	hard & very hard (min/d)	**r = .639, p = .000**	r = .233, p = .143	**r = .558, p = .000**

HAES	active (min/d)	**r = .403, p = .009**	r = .101, p = .528	**r = .326, p = .037**
	
	somewhat active & active (min/d)	r = .092 p = .566	r = .003, p = .983	r = -.102, p = .522

LRC	activity category	r = -.056, p = .728	r = .017, p = .971	r = -.007, p = .965

Values are Pearson product correlation coefficients (HAES; 7D-PAR) or Spearman rank correlation coefficients (LRC), and the respective probability of a type I error (p). For further abbreviations, see legend Table 2.

Significant correlations were observed between MPA and MVPA measured by accelerometry and the activity categories 'hard', 'very hard', 'moderate & hard & very hard', and' hard & very hard activity' reported in the 7D-PAR. For VPA a significant correlation was only evident with the category 'very hard' of the 7D-PAR. The category'active' of the HAES also showed a moderate but significant correlation with MPA and MVPA, but not with VPA. There was no association between physical activity measured by accelerometry and the individual's activity level determined by the LCR.

The results of the exercise test (Wmax and VO_2_peak) did not correlate with the results of the HAES and 7D-PAR (data not shown). However, the individuals' activity levels derived from the LRC showed a moderate correlation with Wmax (r = 0.46, *p *= 0.002) and a weak correlation with VO_2_peak (r = 0.32, *p *= 0.041).

The ICC-analyses of the accelerometer data and the data derived from the 7D-PAR and HAES (Table 4) revealed that only the 7D-PAR categories 'hard', 'very hard', and 'hard & very hard' significantly reflected objectively measured PA. None of the HAES activity categories showed significant ICCs with the PA categories determined by accelerometry.

**Table 4:** Intraclass correlation coefficients (ICC) relating measures of PA derived from the 7D-PAR and the HAES questionnaires to PA measured by accelerometry

		MPA (min/d)	VPA (min/d)	MVPA (min/d)
7D-PAR	moderate (min/d)	ICC = .066, p = .338	ICC = .097, p = .374	ICC = -.013, p = .532

	hard (min/d)	**ICC = .448, p = .001**	ICC = .071, p = .328	**ICC = .443, p = .002**

	very hard (min/d)	**ICC = .306, p = .024**	**ICC = .267, p = .044**	**ICC = .408, p = .004**

	Moderate & hard & very hard (min/d)	ICC = .114, p = .235,	ICC = .020, p = .449	ICC = .132, p = .203

	hard & very hard (min/d)	**ICC = .337, p = .015**	ICC = .107, p = .250	**ICC = .404, p = .004**

HAES	active (min/d)	ICC = .147, p = .177	ICC = .032, p = .421	ICC = .171, p = .292,

	somewhat active & active (min/d)	ICC = .023, p = .442	ICC = -.022, p = .555	ICC = .001, p = .497

For abbreviations, see legend Table 2.

Significant correlations were observed between MPA and MVPA measured by accelerometry and the activity categories 'hard', 'very hard', 'moderate & hard & very hard', and' hard & very hard activity' reported in the 7D-PAR. For VPA a significant correlation was only evident with the category 'very hard' of the 7D-PAR. The category'active' of the HAES also showed a moderate but significant correlation with MPA and MVPA, but not with VPA. There was no association between physical activity measured by accelerometry and the individual's activity level determined by the LCR.

The results of the exercise test (Wmax and VO_2_peak) did not correlate with the results of the HAES and 7D-PAR (data not shown). However, the individuals' activity levels derived from the LRC showed a moderate correlation with Wmax (r = 0.46, *p *= 0.002) and a weak correlation with VO_2_peak (r = 0.32, *p *= 0.041).

The ICC-analyses of the accelerometer data and the data derived from the 7D-PAR and HAES (Table 4) revealed that only the 7D-PAR categories 'hard', 'very hard', and 'hard & very hard' significantly reflected objectively measured PA. None of the HAES activity categories showed significant ICCs with the PA categories determined by accelerometry.

**Figure 1:**
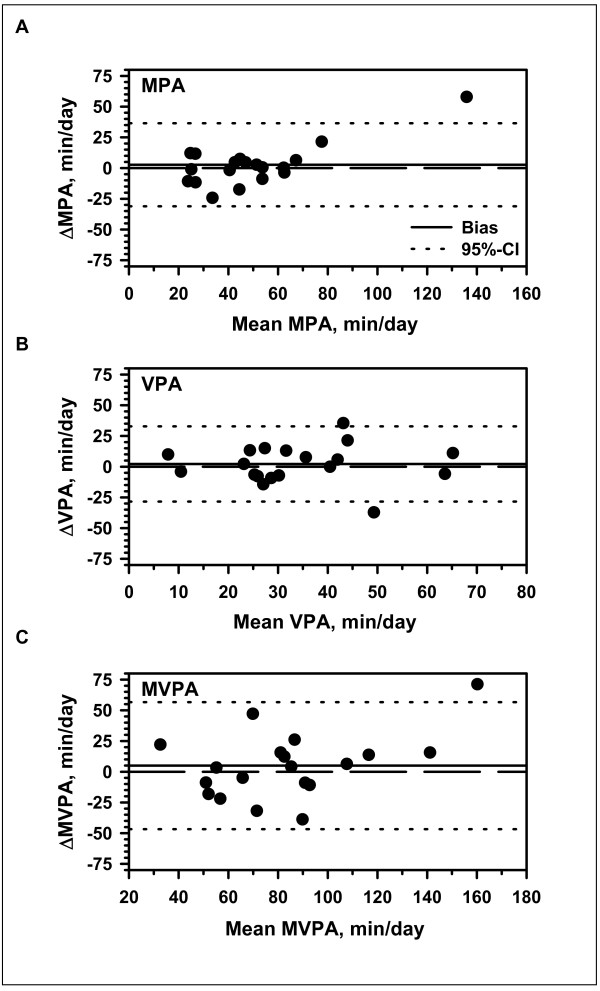
**Bland-Altman plots of the test-retest accelerometry data for each activity category; A: Moderate physical activity (MPA), B: Vigorous physical activity (VPA) C: Moderate and vigorous physical activity (MVPA)**.

A test-retest analysis in the subgroup of 19 participants showed a moderate to strong reproducibility of the time an individual patient spent in the accelerometer categories, MPA, VPA, and MVPA with higher ICCs for MPA and MVPA than for VPA (MPA: ICC = 0.804, *p *= 0.000; VPA: ICC = 0.578, *p *= 0.004; MVPA: ICC = 0.702, *p *= 0.000). A Bland-Altman plot (see Figure 1) further illustrates the test-retest analysis of the three categories MPA, VPA, and MVPA. Limits of agreement were, 56.6/-46.8 min/day for MPA, 32.9/28.4 min/dayfor VPA and 36.4/-31.1 min/day for MVPA.

## Discussion

The aim of this study was to evaluate questionnaires assessing physical activity by comparing them to objectively measured physical activity. Measures of physical activity derived from the 7D-PAR as well as the HAES showed significant correlations to physical activity assessed by accelerometry. In the validity analyses based on ICCs, though, only the physical activity calculated from the 7D-PAR categories 'hard', 'very hard' and "hard & very hard" significantly reflected objectively measured physical activity. To our knowledge, this study is the first to validate the 7D-PAR against accelerometry in a group of patients with CF.

With regard to the HAES, we were able to reproduce the significant Pearson product correlations reported by Wells et al. [13]. However, in contrast to our findings (Table 4), Wells et al. [13] found a significant ICC of 0.44 by relating accelerometry data to the 'total activity' assessed by the HAES, which combined the categories 'somewhat active' and 'very active' in a group of 14 rather healthy (FEV1 > 70%pred) adolescent patients with a mean age of 16 years.

Comparing questionnaires to accelerometers implies that the intensity of activities within each category is comparable between the two modes. According to the ACSM guidelines [9], MPA equals 3.0-6.0 metabolic units (MET) and VPA is seen as an activity > 6.0 METs; thus MVPA would be defined as an activity requiring more than 3 METs. In the 7D-PAR, the activity category 'moderate' reflects 3.0 to 4.9 METs, 'hard' equals 5.0 to 6.9 MET and the category 'very hard' represents 7 or more METs [11]. In the description of the HAES, no MET values are mentioned for the corresponding activity levels. When comparing the examples given to help the participants to categorize their activities to the Compendium of Physical Activities [9], which defines MET intensities for the respective activities, it seems that MVPA is solely represented by the category 'very active'. By combining the categories 'somewhat active' and 'very active' of the HAES (=total activity) light activities are included which should result in a higher amount of physical activity than MVPA determined by accelerometry. This is exactly what we found (Table 2) and might explain why there were no significant ICCs between total activity determined from the HAES and MVPA measured by accelerometry. Furthermore, in contrast to the HAES, the 7D-PAR asks for separate reporting of moderate, hard and very hard physical activity. Therefore the 7D-PAR may allow a better recollection of activities leading to more useful data when compared to accelerometry.

Chinapaw et al. [23] as well as Boon et al. [24] showed that questionnaires tend to overestimate activity time in a certain category in comparison to accelerometry in healthy adolescents and adults. This finding was reproduced in our study in patients with CF with regard to the HAES and the 7D-PAR. Nonetheless, the difference between questionnaire-reported and accelerometer-assessed physical activity may have other reasons. Trost et al. [25] demonstrated that not every activity can be adequately recorded by accelerometry and that especially free-living activities are underestimated in their energy expenditure and their intensity by accelerometry. This is especially true for physical activity predominantly performed by the upper body and physical activity including gliding activities on a vehicle [26]. Hence, the difference of daily habitual physical activity between questionnaires and accelerometers might seem larger in the analysis than it actually is in real life.

None of the physical activity questionnaires (7D-PAR, HAES, and LRC) used in this study was able to detect the gender difference in MVPA measured by accelerometry which has also been previously reported for healthy individuals and those with CF [1].

In patients with CF, regular physical activity is linked to a better lung health, physical fitness, nutritional status and quality of life [3,5,27,28]. Monitoring physical activity is thus important to identify patients who may benefit from activity interventions. As our results showed, only the 7D-PAR and the HAES were able to reflect objectively measured physical activity. However, these questionnaiers are not measuring physical activity precise enough to obtain data which could be used for individual counselling. Thus, on an individual basis, other means such as accelerometry are required.

Whereas the LRC activity levels did not reflect PA measured by accelerometer, a moderate correlation was observed with Wmax and VO_2_peak. This finding is in line with the original validation study of the LRC which showed a significant association of the LRC activity levels with VO_2_peak but not with physical activity measured by accelerometry in a healthy adult population [8,29,30]. In contrast to the individual LRC activity levels, neither the physical activity derived from the 7D-PAR or from the HAES questionnaire correlated with measures of physical fitness. Other studies have shown some positive relationship between VO_2_peak and PA determined by the 7D-PAR in adults, although the association was not always significant (for example [31]).

Hebestreit et al. [4] showed that objectively measured physical activity is related to aerobic fitness which is related to survival in CF [5]. At least some of the effects of physical activity on health in CF may be moderated by an increase in aerobic fitness. Here, vigorous activities are most effective. The inability of the 7D-PAR and the HAES to reflect aerobic fitness may be attributed to the fact that vigorous activities were not captured as well as moderate activities (Tables 3 and 4). Although the activity level derived from the LRC questionnaire was correlated with aerobic fitness, the association was not striong enough to be informative on an individual level. The gold standard for determining aerobic fitness is the measurement of VO_2_peak.

There are some limitations of the present study. First, the accelerometry was performed 2-6 weeks prior to completing the questionnaires. This approach might have weakened the observed relationships between physical activity measured by accelerometry and physical activity assessed by questionnaires. However, as shown in the test-retest analysis and in Figure 1, there was a high consistency of activity behaviour suggesting high stability of physical activity behaviour over a period of 2-6 weeks. Furthermore, as patients with CF are typically seen at their CF centre in intervals of 4-12 weeks the schedule of data collection best reflects the situation of centre care where physical activity may be assessed in intervals of at least 3 months. Second, in contrast to other studies, the 7D-PAR was not interviewer-administered in our study but employed as questionnaire. However, one investigator was always present during the completion of questionnaires and ready to answer any question raised by the participants. This rationale was chosen as we wanted to identify and test questionnaires to be used in a CF centre setting, where interview-administered questionnaires would probably not be useful for an every day usage. After filling in the questionnaire, the investigators checked the questionnaires for plausibility of the answers given. Third, the majority of the participants included in our study were in a relatively good clinical condition (Table 1). Likewise, the proportion of adult participants (i.e. 25%) in our sample was relatively small. Fourth, we used the cut-offs established by Hebestreit et al. [4,6] to determine activity categories. Within accelerometer research a wide variety of cut-offs has been published. When using different cut-offs, the ICC results may also change. Fifth, due to the translation of the questionnaires into German, cultural and translational matters may result in a slightly different understanding of some questions.

## Conclusions

In summary, the information on a patient's physical activity derived from questionnaires is hampered by only moderate relationships to the accelerometer measurements. The highest validity based on ICC analyses was observed for the 7D-PARs categories 'hard', 'very hard' and 'hard & very hard', and the latter showed the strongest Pearson product correlation with MVPA. Therefore, the summed categories 'hard & very hard' might be best suited to describe physical activity in patients with CF. However, even the 7D-PAR could provide only rough estimates of an individual's objectively measured physical activity. None of the physical activity questionnaires was precise enough to be useful for individual counselling. For this purpose, additional objective measures such as accelerometry are required. For epidemiological studies and registries, however, a physical activity questionnaire might be sufficient to describe a CF population's activity behaviour. Further studies should explore the generalizability of our findings by including a more heterogeneous CF population, i.e. more adults and more participants with severe disease.

Abbreviations: 7D-PAR, seven day physical activity recall questionnaire; CF, cystic fibrosis; FEV1, forced expiratory volume in 1 second; FVC, forced vital capacity; HAES, habitual activity estimation scale; ICC, intraclass correlation coefficient; LRC, lipid research clinics questionnaire; MET, metabolic unit; MPA, moderate physical activity; MVPA, moderate to vigorous physical activity; RV, residual volume; TLC, total lung capacity; VPA, vigorous physical activity

## Competing interests

The authors declare that they have no competing interests

## Authors' contributions

KCR, SK, KR, AM and HH designed the study. KCR, SF, MB, AM, and HH collected and analyzed the data. The manuscript has been drafted by KCR and was critically revised by KR, AM, SK and HH. All authors read and approved the final manuscript.

## Pre-publication history

The pre-publication history for this paper can be accessed here:

http://www.biomedcentral.com/1471-2288/12/43/prepub

## Supplementary Material

Additional file 1 **Detailed description of physical activity questionnaires**. Click here for file
