# Supplementary material for: Validation of activity questionnaires in patients with cystic fibrosis by accelerometry and cycle ergometry
Source: BMC Med Res Methodol. 2012 Apr 3;12:43. doi: 10.1186/1471-2288-12-43 (PMC3359253; doi:10.1186/1471-2288-12-43)
Supplement: Additional file 1 — Detailed description of physical activity questionnaires. [file 1471-2288-12-43-S1.DOCX]

# Additional files

Additional file 1 – **Detailed description of physical activity questionnaires**

Seven Day Physical Activity Recall questionnaire (7D-PAR)

The 7D-PAR asks the patients to recall the time they spent at different activity intensities during the last 7 days. Patients are asked to report the daily time spent sleeping (1 metabolic unit = MET), as well as the hours spent with ‘moderate‘ (3-4.9 METs), ‘hard‘(5.0-6.9 METs) and ‘very hard‘ (≥7 METs) activities separately for the five weekdays and the two weekend days [O1, O2]. To help with the classification of activities, several examples are given in the questionnaire for each activity category; ‘moderate activities’ such as raking the lawn; sweeping and mopping; cleaning windows, volleyball, ping-pong, brisk walking, golf; ’hard activities’ such as scrubbing floors, tennis doubles, dancing; and ’very hard activities’ such as carrying heavy loads, jogging or swimming, single tennis, soccer. In order to calculate the amount of time spent in a given activity category during the respective week, the respective data from weekdays and weekend days were added and then divided by 7.

Habitual Activity Estimation Scale (HAES)

The HAES questionnaire asks for the percentage of time spent within four different activity levels (’inactive’, ’somewhat inactive’, ‘somewhat active’, and ‘very active’) on a typical weekday of the previous week (Tuesday, Wednesday, or Thursday) and on the previous Saturday [O3]. Patients can classify their activities with the help of examples given for each activity level (e.g. inactive: lying down, sleeping, resting, napping; somewhat inactive: reading, watching television, playing video games, time in front of the computer; somewhat active: walking, shopping, light household chores; very active: running, jumping, skipping, bicycling, skating, swimming). The mealtimes, wake-up time and bedtime reported by the patient allowed us to calculate the minutes per day spent within each activity category for the respective periods of time. Minutes per day within the respective week were computed by taking the reported weekday time times 5 and the weekend time by 2 and then dividing the sum of both by 7.

Lipid Research Clinics questionnaire (LRC)

From the LRC two questions determined the physical activity status of the participants: one question asked for engagement in regular strenuous exercise or labour (yes or no), the other assessed the individual’s perceived level of physical activity engagement compared to peers of the same age and sex. Those who reported regular strenuous exercise or labour were classified as ’moderately active’ or ‘highly active’ depending on their answer to the second question. Those who were not regularly physically active were classified as ‘low active’ or ‘very low active’ depending on their rating in the second question (see Fig. O1) [O4]. The resulting activity level was expressed as a point value between 1 and 4, 1 being ‘very low active’ and 4 being ‘highly active’.

|  | | **Self rating of physical activity during leisure compared to peers** | | |
| --- | --- | --- | --- | --- |
|  |  | *A lot more* | *A little more/*  *about the same* | *A little less/*  *much less* |
| **Strenuous exercise or labour** | *yes* | High active  (Category 4) | Moderately active  (Category 3) | |
|  | *no* | Low active  (Category 2) | | Very low active (Category 1) |

Figure O1 – classification of activity level by the LRC

O1. Sallis JF, Buono MJ, Roby JJ, Micale FG, Nelson JA. Seven-day recall and other physical activity self-reports in children and adolescents. *Med Sci Sports Exerc* 1993; **25**: 99-108.

O2. Sallis JF, Haskell WL, Wood PD, Fortmann SP, Rogers T, Blair SN, Paffenbarger RS Jr. Physical activity assessment methodology in the Five-City Project. *Am J Epidemiol* 1985; **121**: 91-106.

O3. Hay J. Development and testing of the habitual activity estimation scale. *In:* Armstrong N, ed. Children and exercise XIX: promoting health and wellbeing. Vol 2. Singer Press, Exeter, 1997; pp. 125-129.

O4. Ainsworth BE, Jacobs DR Jr., Leon AS. Validity and reliability of self-reported physical activity status: the Lipid Research Clinics questionnaire. *Med Sci Sports Exerc* 1993; **25**: 92-98.
